# A computationally driven analysis of the polyphenol-protein interactome

**DOI:** 10.1038/s41598-018-20625-5

**Published:** 2018-02-02

**Authors:** Sébastien Lacroix, Jasna Klicic Badoux, Marie-Pier Scott-Boyer, Silvia Parolo, Alice Matone, Corrado Priami, Melissa J. Morine, Jim Kaput, Sofia Moco

**Affiliations:** 1 The Microsoft Research – University of Trento Centre for Computational and Systems Biology (COSBI), Rovereto (TN), Italy; 2 Nestle Institute of Health Sciences, Lausanne, Switzerland; 3 Department of Computer Science, University of Pisa, Pisa (PI), Italy; 4 Present Address: Institute of Nutrition and Functional Foods (INAF), Québec, Canada; 5 Present Address: Centre de Recherche du Centre Hospitalier Universitaire de Québec (CRCHUQ), Québec, Canada

## Abstract

Polyphenol-rich foods are part of many nutritional interventions aimed at improving health and preventing cardiometabolic diseases (CMDs). Polyphenols have oxidative, inflammatory, and/or metabolic effects. Research into the chemistry and biology of polyphenol bioactives is prolific but knowledge of their molecular interactions with proteins is limited. We mined public data to (i) identify proteins that interact with or metabolize polyphenols, (ii) mapped these proteins to pathways and networks, and (iii) annotated functions enriched within the resulting polyphenol-protein interactome. A total of 1,395 polyphenols and their metabolites were retrieved (using Phenol-Explorer and Dictionary of Natural Products) of which 369 polyphenols interacted with 5,699 unique proteins in 11,987 interactions as annotated in STITCH, Pathway Commons, and BindingDB. Pathway enrichment analysis using the KEGG repository identified a broad coverage of significant pathways of low specificity to particular polyphenol (sub)classes. When compared to drugs or micronutrients, polyphenols have pleiotropic effects across many biological processes related to metabolism and CMDs. These systems-wide effects were also found in the protein interactome of the polyphenol-rich citrus fruits, used as a case study. In sum, these findings provide a knowledgebase for identifying polyphenol classes (and polyphenol-rich foods) that individually or in combination influence metabolism.

## Introduction

Intake of polyphenols has been associated with many health benefits and these compounds are the most known and widely studied class of plant natural compounds. Polyphenols are defined as secondary metabolites resulting from the shikimate pathway-derived phenylpropanoid and/or the polyketide pathway(s)^1^. Polyphenols have more than one phenolic ring and are devoid of any nitrogen-based functional group in their most basic structural expression^1^. However, ‘polyphenol’ is also commonly used for compounds that may contain only one phenolic ring such as phenolic acids, which do not meet the described chemical definition. As broadly defined, polyphenols account for more than 80,000 known compounds with molecular masses up to 30,000 Da (e.g., tannins)^2^.

Polyphenols are widely distributed among higher plants and thus abundant in plant-based diets. Fruits, vegetables, legumes, cereals, and beverages such as tea, coffee, wine, and beer are rich sources of polyphenols. The polyphenol content in certain foods can surpass 1 g of total polyphenols/100 g of food material with cocoa powder as a prime example^3^. In spite of this relatively high content in food, the bioavailability of polyphenols is often limited since they are largely metabolized by the gut microbiota as well as the host^4,5^. Developing knowledge of the food polyphenol metabolome^6^ – that is, the ensemble of metabolites found in the body derived from polyphenols or polyphenol-rich food consumption - is crucial to understand the role of this class of bioactives in metabolism, activity, and health.

Fruit and vegetable intake is strongly associated with reduced risk of cardiovascular disease, cancer, and all-cause mortality^7^. A daily intake of 200 g (or 2 ½ portions) of fruits and vegetables was associated with an 8–13% reduction in risk of cardiovascular disease. The predicted reduction of risk would be 28% if the recommended daily intake would increase to 800 g/day (or 10 daily portions). Apples, pears, citrus fruits, cruciferous vegetables, green leafy vegetables, tomatoes, β-carotene-rich and vitamin C-rich fruit and vegetables were found to be best at preventing coronary heart disease and stroke^7^.

The reduced CMD risk associated with fruits and vegetables has been assumed to be due to various specific compounds, among these are polyphenol content and composition^8^. Polyphenols have been associated with various health benefits based on their possible antioxidant capacity^1^. However, this concept has generally been abandoned given the strong evidence that polyphenols can specifically interact with protein targets irrespective of their redox properties and thereby modulate signalling and metabolic pathways relevant in cardiovascular^9,10^ and neurodegenerative^11^ diseases, as well as cancer^12^ and diabetes^13^. Widely studied polyphenols such as resveratrol, curcumin, epigallocatechin-3-gallate, and quercetin have been associated with multiple protein targets and pathways, with potential therapeutic applications for a myriad of diseases. These natural compounds have been described as important leads for multi-target drug development^14,15^.

Claims have been made that polyphenols have beneficial effects for management of type 2 diabetes and metabolic syndrome through various mechanisms^16,17^. For example, flavonoids have shown inhibitory activity for α-glucosidase^18^, a known molecular target for diabetes. The green tea polyphenol epigallocatechin-3-gallate inhibits gluconeogenesis by activating 5′-AMP-activated protein kinase (AMPK) through Ca^2+^/calmodulin-dependent protein kinase kinase (CaMKK)^19^. Resveratrol has also been shown to stimulate AMPK which is mediated by the NAD-dependent deacetylase sirtuin-1 (SIRT1), consequently improving mitochondrial function *in vivo*^20^.

Given the wide range of effects reported for polyphenols, we mapped the polyphenol-protein interactome by surveying public knowledge to provide a systems-wide overview of state-of-the-art of polyphenol research. Our approach was to determine the full known polyphenol (and human polyphenol-derived metabolites)-protein interactome by chemically defining the polyphenol space, mining public available databases for protein interactions, and performing functional analysis through mapping interacting proteins to metabolic pathways. Our results link the specificity but also pleotropic metabolic effects of this class of compounds with processes involved in CMDs. Micronutrients- and metformin- protein interactomes were used as a comparison of the extent of effects of the polyphenol-protein interactome. To test our computational strategy for nutritional impact, lemon, orange, lime, grapefruit, tangerine and pomelo citrus fruits were compared for polyphenol content, protein interactome, and pathway enrichment.

## Results

### Chemically defining polyphenols

Polyphenols occupy a wide chemical space. For the purposes of this study, polyphenols were defined according to structural features of classes and subclasses as classified by Phenol-Explorer^21^. This food polyphenol database categorizes polyphenols into 6 classes (flavonoids, phenolic acids, lignans, stilbenes, other polyphenols, and non-phenolic metabolites of polyphenols) with a total of 31 sub-classes (e.g. flavones, hydroxybenzoic acids, alkylphenols).

**Figure 1:**
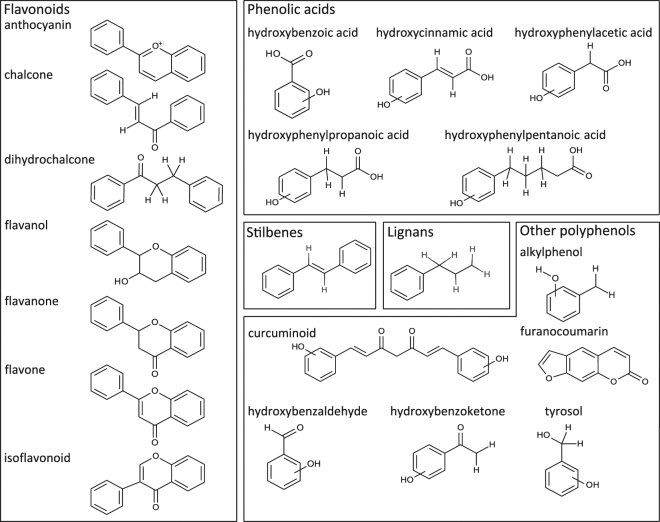
Forty-three distinct substructures of polyphenols produce 5 classes: (**i**) flavonoids (9 subclasses: anthocyanins, chalcones, dihydrochalcones, dihydroflavonols and flavanone, flavone and flavonol, isoflavonoid), (**ii**) lignans, (**iii**) phenolic acids (hydroxybenzoic acids, hydroxycinnamic acids, hydroxyphenylacetic acids, hydroxyphenylpropionic acids, hydroxyphenylpentanoic acids), (**iv**) stilbenes, and (**v**) other polyphenols (alkylmethoxyphenols and hydroxyphenylpropenes and alkylphenols, curcuminoids, furanocoumarins, hydroxybenzaldehydes and hydroxycinnamaldehydes, hydroxybenzoketones, tyrosols). Some polyphenol subclasses have several scaffolds (e.g., phenolic acids) to describe ortho-, para-, and meta- substitutions, while several polyphenol subclasses (e.g., flavonols and flavones) have a single substructure. Some substructures include more than one subclass (redundancy). Non-polyphenolic metabolites (6^th^ class) could not be queried, as not having fixed structural features.

Polyphenols and polyphenol metabolites retrieved from Phenol-Explorer contained 752 unique structures. To widen the number of searchable polyphenols, the Phenol-Explorer polyphenol classification was used to define 43 different chemical substructures (Fig. 1) characteristic of polyphenols. These substructures were used to query, by structural features, the Dictionary of Natural Products, DNP^2^, a comprehensive structural database of natural products. This query resulted in a total of 36,064 unique polyphenols. This combined list of polyphenols from these two sources (Phenol-Explorer and DNP) was used for mining protein interactions databases.

### Polyphenol-protein interactome

The CHEBI database was searched for unique identifiers for all listed polyphenols. The search was based on canonical SMILES strings of standardized structures and required strict match of stereochemistry. The result of the CHEBI search yielded 1,395 unique polyphenols (1,032 from DNP and 222 from Phenol-Explorer), which was less than 4% of the original 36,064 unique polyphenols (Table 1S).

**Table 1:** Representative proteins (gene names) that interact with polyphenols.

Protein class	Number of interacting proteins	Total number and (unique polyphenols)
**Detoxification enzymes**		
Cytochrome P450 (CYPs)	44	249 (87)
UDP-glucuronyl transferases (UGTs)	21	162 (36)
Aldehyde dehydrogenase (ALDHs)	18	80 (25)
Carbonic anhydrases (CAs)	12	301 (69)
Aldo-keto reductases (AKRs)	11	162 (87)
Sulfotransferases (SULTs)	9	30 (17)
Glutathione *S*-transferases (GSTs)	9	26 (16)
Alcohol dehydrogenase (ADHs)	6	12 (5)
Cysteine conjugate *N*-acetyltransferases (NATs)	5	5 (2)
Phosphodiesterases (PDEs)	5	17 (15)
Glutathione peroxidase (GPxs)	4	12 (9)
Monoamine oxidases (MAOs)	2	92 (58)
Catechol-*O*-methyltransferase (COMTs)	2	24 (23)
Carbonyl reductase (NADPH) (CBRs)	2	8 (8)
Aldehyde Oxidases (AOXs)	1	17 (17)
D-amino acid oxidases (DAO)	1	3 (3)
Flavin-containing monooxygenases (FMOs)	1	1 (1)
Quinone reductases (CRYZ)	1	1 (1)
Epoxide hydrolases (EHs)	1	1 (1)
**Metabolism enzymes**		
Hydroxysteroid dehydrogenase (HSDs)	12	108 (62)
Prostaglandin-endoperoxide synthase (PTGs)	11	84 (51)
Lipoxygenases (ALOXs)	7	195 (102)
NADPH oxidases (NOXs)	2	48 (48)
Xanthine dehydrogenase (XDH)	1	61 (61)
**Membrane transport proteins**		
Solute carrier family (SLC)	145	265 (46)
ATP-binding cassette transporters (ABCs)	22	208 (95)
Proton-translocating cytochrome oxidases (COX)	6	7 (5)
Acyl-CoA thioesterases (ACOT)	5	7 (4)
GABA transporters (GAT)	5	6 (4)
Monoamine transporters (MAT)	3	3 (2)
Major facilitator superfamily (MFS)	3	3 (1)
Fatty acid transporter (FAT)	3	3 (2)
**Nuclear receptors**		
Peroxisome proliferator-activated receptor (PPARs)	5	27 (19)
3-Ketosteroid receptors (NR3Cs)	3	38 (27)
Retinoid X receptor (RXRs)	3	9 (8)
Liver X receptor-like (NR1Hs)	3	4 (3)
Nerve Growth Factor IB-like (NR4As)	3	4 (4)
Testicular receptor (NR2Cs)	3	3 (2)
Oestrogen related receptors (ERs)	2	130 (87)
Retinoic acid receptor (RARs)	2	19 (14)
Thyroid hormone receptor (THRs)	2	18 (9)
Vitamin D receptor-like (NR1Is, VDR)	2	15 (12)
Dosage-sensitive sex reversal / small heterodimer partner (DAX/SHP, NR0Bs)	2	4 (4)
Chicken ovalbumin upstream promoter transcription factor (COUP/EAR, NR2Fs)	2	2 (1)
Reverse erbA (Rev-ErbA, NR1Ds)	1	1 (1)
Hepatocyte nuclear factor-4 (HNF4s)	1	1 (1)
**G-Protein coupled receptors**		
Rhodopsin-like receptors	64	178 (77)
Secretin receptor family	5	8 (7)
Metabotropic glutamate/pheromone receptors	6	10 (6)
Frizzled/Smoothened receptors	3	4 (4)
**Other receptors**		
Tumor necrosis factor (receptors) (TNFs)	19	95 (61)
**ATP binding**		
Myosin (MYHs, MYLs, MYOs)	28	39 (6)
Phosphoribulokinase/uridine kinase (PRKs)	26	51 (14)
Cyclin-dependent kinases (CDKs)	23	119 (34)
Mitogen-activated protein kinases (MAPKs)	11	153 (62)
Ca^2+^/calmodulin-dependent protein kinase (CAMKs)	8	11 (4)
Casein kinases (CKs)	6	13 (8)
A-kinase anchoring proteins (AKAPs)	3	4 (4)
Aurora kinases (AURKs)	2	30 (26)
Protein kinases B (AKTs)	2	26 (24)
Glycogen synthase kinase 3 (GSK-3s)	2	16 (13)
Meiotic checkpoint protein kinases (CHEKs)	2	9 (6)
**DNA binding**		
Histones (HISTs)	47	59 (6)
Signal transducer and activator of transcription proteins (STATs)	7	27 (15)
Poly (ADP-ribose) polymerase (PARPs)	4	30 (27)
Nuclear factor kappa-light-chain-enhancer of activated B cells (NF-kB)	4	27 (19)
**Diabetes**		
AMP-activated protein kinase (PRKAs, PRKBs, PRKGs)	7	11 (2)
Peroxisome proliferator-activated receptor (PPARs)	5	27 (19)
Histone deacetylases (HDAC)	5	6 (4)
Glycogen phosphorylase (PYGB, PYGL, PYGM)	3	8 (4)
Inhibitor of nuclear factor kappa-B kinase (IKBKB, CHUK)	2	7 (7)
Sodium glucose co-transporter 2 (SLC5A2)	1	6 (6)
Free fatty acid receptor 1 / GPR40 (FFAR1)	1	4 (4)
Dipeptidyl peptidase 4 (DDP-4)	1	4 (4)
X-box binding protein 1 (XBP1)	1	3 (3)
Insulin receptor (INSR)	1	2 (2)
Alpha-glucosidase (GAA)	1	2 (2)

**Figure 2:**
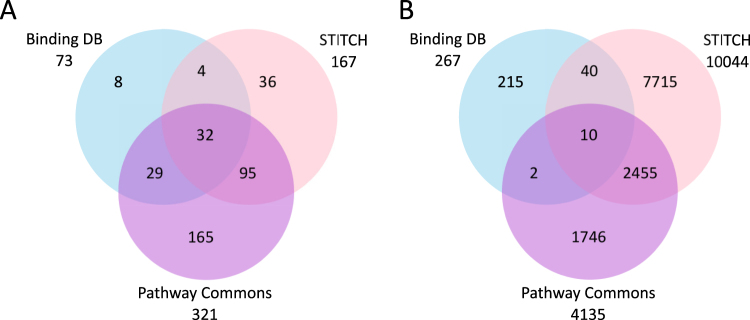
(**A**) Distribution of polyphenols and (**B**) polyphenol interacting proteins among protein interaction databases (BindingDB, STITCH and Pathway Commons).

The knowledge-bases STITCH^22^ and Pathway Commons^23^ contain small molecule-protein interaction information based on 23 source interaction databases. By using these 2 knowledge bases and the small molecule-protein direct interaction database BindingDB^24^, known public information was found for human protein interactions for the 1,395 unique polyphenols. A total of 11,987 polyphenol-protein interactions for 369 unique polyphenols and their metabolites (26% of all compounds) with 5,699 unique interacting proteins were identified. The majority of the interacting polyphenols were from DNP (198 unique polyphenols), compared to Phenol-Explorer (83 unique polyphenols), and some were found in both databases (88). Flavones, hydroxybenzoic acids, and alkylphenols are the sub-classes with the largest number of interactions with proteins (Fig. 1S). Pathway Commons contains the most diverse number of interacting polyphenols (>300 - Fig. 2A), while STITCH contains most of the interactions (>10,000 - Fig. 2B). Remarkably, only a few polyphenols and proteins were present in more than one interaction database (Fig. 2). For example, BindingDB does not contain any protein interactions with stilbenes or lignans while STITCH provided most of the interaction information for flavonoids (80% of all flavonoids).

**Figure 3:**
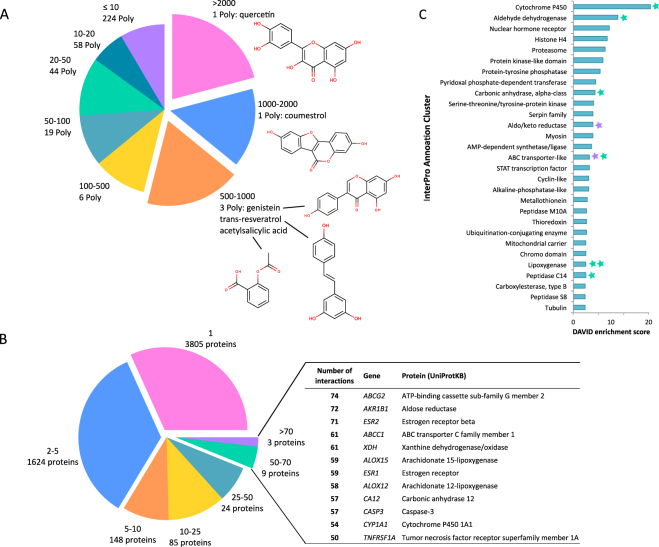
(**A**) Distribution of polyphenol-protein interactions (polyphenols interacting with: >2000, 1000–2000, 500–1000, 100–500, 50–100, 20–50, 10–20, and ≤10 proteins) and associated number of polyphenols (Poly). (**B**) Distribution of polyphenol-protein interactions (proteins interacting with: >70, 50–70, 25–50, 10–25, 5–10, 2–5, and 1 polyphenols) and associated number of proteins. Proteins interacting with >50 polyphenols: gene name, corresponding protein name (according to UniProtKB) are noted. (**C**) DAVID annotation clustering of polyphenol interacting proteins with enrichment score >3, using InterPro classification. Proteins with >70 (violet) and 50–70 (green) interactions belonging to InterPro annotation clusters are indicated.

Only a few compounds account for the majority of interactions found in the interactome databases. More than half of the identified polyphenol-protein interactions belong to quercetin (2,500 interactions), coumestrol (1,802 interactions), genistein (916 interactions), trans-resveratrol (738 interactions), and acetyl-salicylic acid (510 interactions), Fig. 3A. Derivatives of quercetin, such as quercetin 3-*O*-β-D-glucopyranoside, quercetin 4′-*O*-β-D-glucopyranoside, and quercetin 3,4′-dimethyl ether, and human metabolically-produced quercetin 3-sulfate show some overlapping protein interactions with quercetin, but in general very few novel interactions were found for polyphenol metabolites (Fig. 2S). Sixty-five percent of the polyphenols have >10 reported polyphenol-protein interactions. Even though quercetin, coumestrol, genistein, trans-resveratrol, and acetylsalicylic acid are reported to have >6,000 found protein interactions, the number of structural data on polyphenol-protein complexes deposited in the Protein Data Bank (PDB)^25^ is <100. Protein interactions with curcumin, also a known polyphenol in the class of ‘Other polyphenols’ (according to Phenol-Explorer), were below STITCH’s evidence score of 0.9, and thus were not selected. Most polyphenol-interacting proteins (95%) interact with <5 polyphenols. A few proteins (12) were found to interact with ≥50 polyphenols, such as ABC-transporters, lipoxygenases, and oestrogen receptors (ERs - Fig. 3B). The polyphenol-interacting proteins were clustered using InterPro^26^ protein superfamily annotations using DAVID^27^. The highest represented functions of polyphenol interacting proteins were drug metabolism (e.g., cytochrome P450s), oxidation (e.g., aldehyde dehydrogenase), cell cycle (e.g., histone H4 and proteasome), regulation of metabolism (e.g., protein kinase-like and protein-tyrosine phosphatase), and nuclear hormone receptors (Fig. 3C). Oestrogen and related receptors and TNF receptor-associated factors (TRAF-like) were poorly enriched (0.2 and 0.9 enrichment scores, respectively). Selected polyphenol-interacting proteins^28–31^ were listed according to cellular functions: detoxification, metabolism, transport, nuclear receptors, and role in diabetes (Table 1).

### Functional pathway analysis of polyphenol-protein interactome

**Figure 4:**
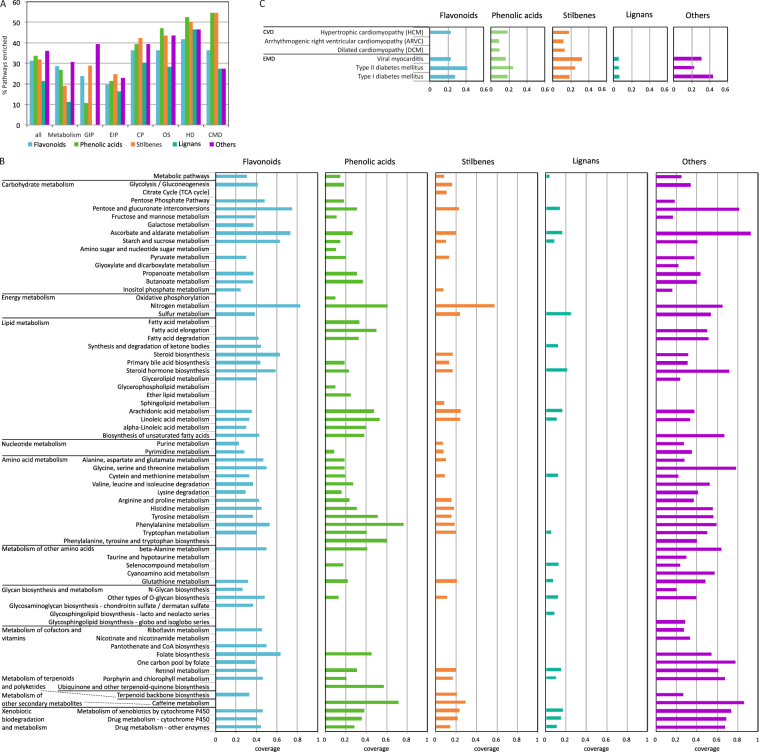
Significant KEGG pathway enrichment by polyphenol class (p-value < 0.1): flavonoids, phenolic acids, stilbenes, lignans, and other polyphenols. (**A**) percentage of KEGG pathways enriched according to KEGG pathway categories Metabolism, GIP (Genetic Information Processing), EIP (Environmental Information Processing), CP (Cellular Processes), OS (Organismal Systems), HD (Human Diseases), and CMD (Cardiometabolic Diseases, as a combination of CVD, Cardiovascular Diseases, and EMD, Endocrine and Metabolic Diseases) for each polyphenol class; (**B**) Coverage of KEGG enriched pathways within the Metabolism category for each polyphenol class; (**C**) Coverage of KEGG enriched pathways with the sub-category CMD (CVD and EMD) for each polyphenol class.

Functional pathway analysis of polyphenol-interacting proteins was done using KEGG^32^ to obtain a systems view of the biological processes affected by polyphenols. Pathway enrichment analyses were performed using polyphenol-protein interactions according to Phenol-Explorer polyphenol classes (flavonoids, phenolic acids, stilbenes, lignans, non-phenolic metabolites, and other polyphenols) with p-values < 0.1 as a cut-off. The significant polyphenols were enriched in 31% (on average) of 480 total KEGG pathways (Fig. 4A). Given this extensive association of polyphenols to proteins involved in many biological functions and pathways, the significantly enriched pathways per polyphenol class were classified using the KEGG sub-network categories: Metabolism, Genetic Information Processing, Environmental Information Processing, Cellular Processes, Organism Systems, and Human Diseases. The strength of enrichment and pathway coverage differed among polyphenol classes and within KEGG categories (Fig. 4). Lignans are enriched in the fewest number of pathways (21%, Fig. 4A) and with the least overall coverage per pathway (<0.23, Fig. 4B). The category Human Diseases is highly enriched (>40%, Fig. 4A) for all polyphenol classes. Within this category, 6 pathways were enriched (i.e. 55%, Fig. 4A) for CMDs (5 pathways related to Cardiovascular Diseases and 6 related to Endocrine and Metabolic diseases), and specifically for phenolic acids and stilbenes. Non-polyphenol metabolites were not enriched in any pathway. The pathway coverage within the Metabolism category was compared for the 5 polyphenol classes (Fig. 4B). Enrichments are found for many pathways related to central metabolism (carbohydrate, energy, amino acids), lipid metabolism, xenobiotic biodegradation, and other metabolism pathways. Nitrogen metabolism is highly enriched for all classes except for lignans. Stilbenes and lignans have the poorest coverage in pathways among all polyphenolic classes. Xenobiotic metabolism is highly enriched in polyphenols belonging to the classes flavonoids, phenolic acids and others. The coverage of pathways related to CMDs (Fig. 4C) indicates that phenolic acids and stilbenes are enriched in 6 of 11 pathways, and flavonoids and other polyphenols have the highest coverage (>0.4) for type 1 and 2 diabetes, respectively.

### Polyphenols effects compared to other bioactives

**Figure 5:**
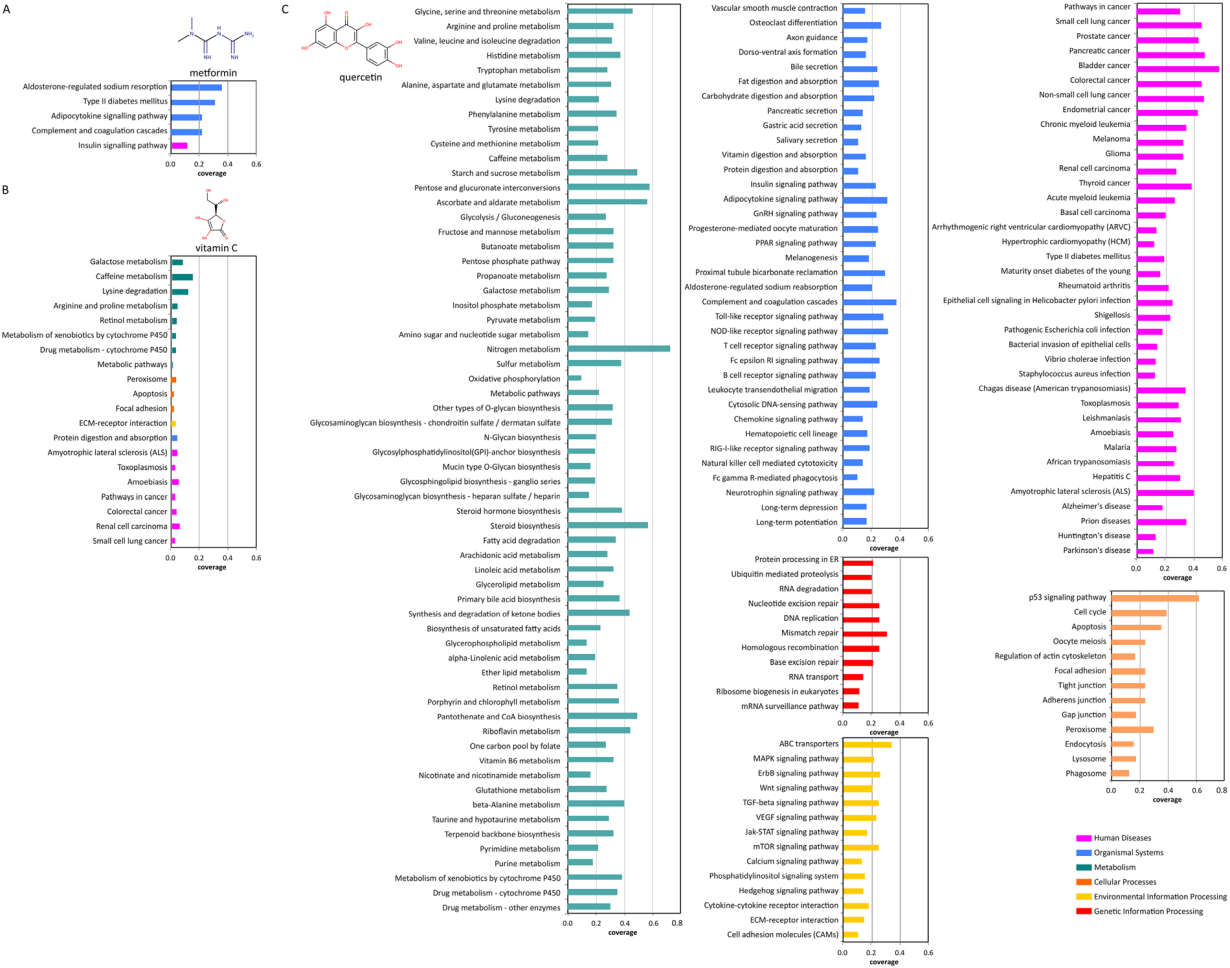
Comparison of significant KEGG pathway enrichments (p-value < 0.1) for metformin (**A**), vitamin C (**B**), and quercetin (**C**).

The small molecule food-derived micronutrients were used as a comparison of the extent of coverage of pathways by polyphenols. Vitamins A, B3, B6, and C were selected because of their involvement in regulation in multiple pathways and health benefits^33^. The number of unique protein interactors found for each vitamin was 50 for vitamins A, C, and B3, and 11 for vitamin B6. Upon pathway enrichment, less than 20 pathways were enriched in a protein interactome of each vitamin and few overlapping pathways between vitamins were found (Fig. 3S, for vitamin C, Fig. 5B). The pathway enrichment of vitamin C was compared to the known polyphenol quercetin (Fig. 5C) and to the known synthetic, bioactive drug, metformin (Fig. 5A). Metformin is widely used in the management of type 2 diabetes^34,35^. The metformin-protein interactome was significantly smaller when compared to most polyphenols (15 interacting proteins), and its pathway enrichment was highly restricted (Fig. 5A).

### Polyphenol-rich food: the case of citrus fruits

**Figure 6:**
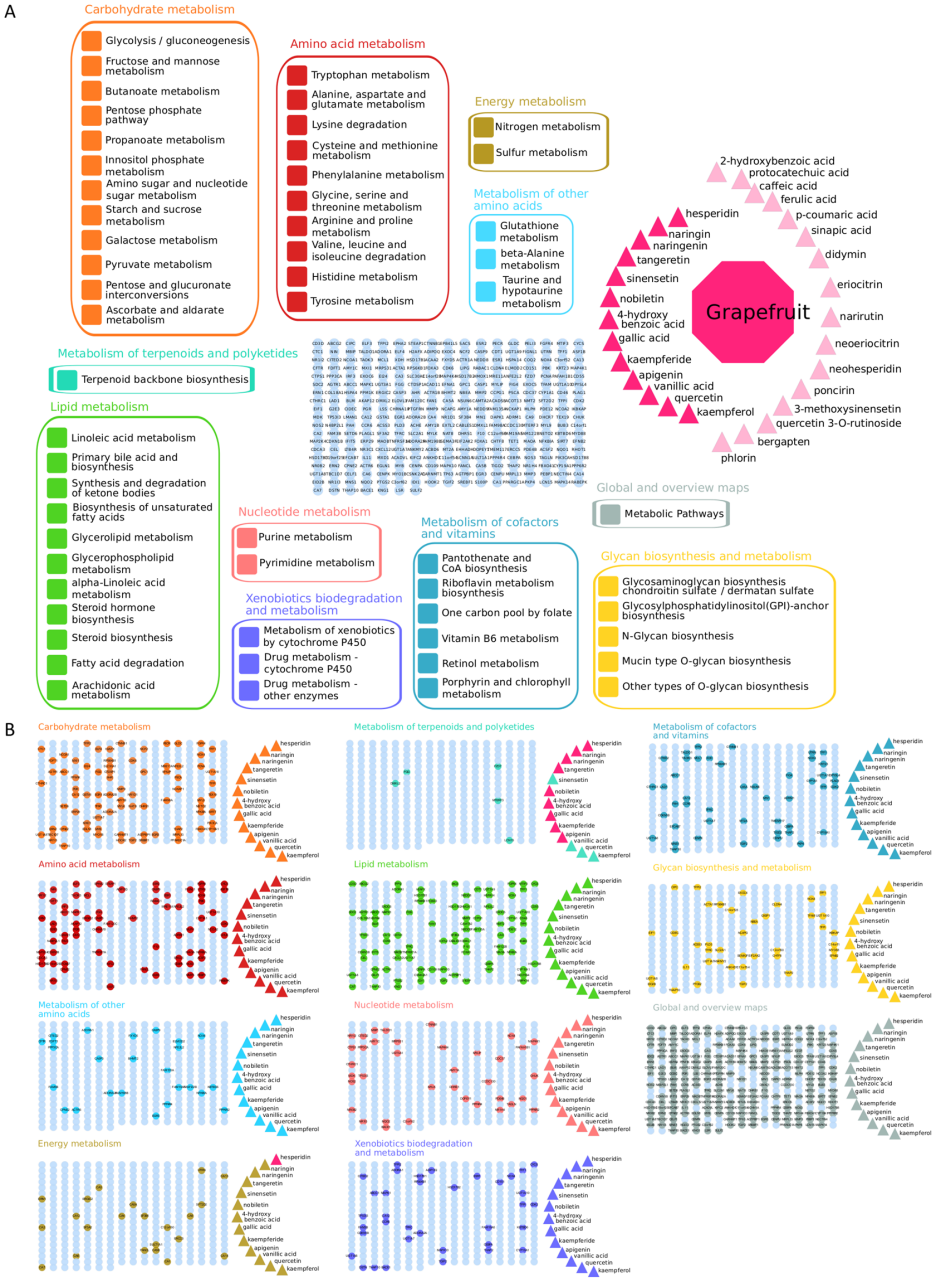
Diagram of metabolic pathway enrichment network for grapefruit polyphenol interactome. (**A**) KEGG Pathway enrichment analysis was performed individually for each grapefruit polyphenol (pink triangles) with known protein interactions. Significant metabolic pathways (BH p-value < 0.1 and pathway coverage >0.2) were represented and color-coded by KEGG sub-pathway categories, and (**B**) corresponding proteins were highlighted for each pathway category.

Citrus fruits were used as an example of polyphenol-rich foods to evaluate the utility of the polyphenol-protein interactome. The polyphenols in grapefruit, lemon, lime, orange, tangerine, and pomelo were compared although lemon and lime contained almost all polyphenols in these 6 fruits. Grapefruit has the most distinct polyphenol composition with an enrichment of phenolic acids. Polyphenols from this citrus class interact with multiple proteins involved in many biological pathways without any fruit containing a unique activity in one pathway or process. Citrus polyphenols are metabolized or interacted with only few pathways in the KEGG’s metabolism category (Fig. 4S). A polyphenol-protein network was built for grapefruit, which highlighted the complexity of biological effects when combining multiple polyphenols present in this citrus, their interacting proteins, and their enriched metabolism pathways (Fig. 6).

## Discussion

Polyphenols are probably the most widely discussed and studied food bioactives in the scientific and popular science worlds. However, misunderstandings are generated in the media and science literature because of the lack of specificity in nomenclature and assumptions of chemical features, which can result in including non-polyphenols as polyphenols. The classification of these compounds is challenging since they are highly diverse with many minor variations in substituents, modifications at different positions, and sub-structures of different chemical classes. In addition, formal nomenclature of natural compounds, such as the one defined by IUPAC (International Union of Pure and Applied Chemistry) is often not practical resulting in use of error-prone, common nomenclature.

A chemical substructure strategy was developed for this report, which defined the polyphenol chemical space by following a classification proposed by the polyphenol-specific database, Phenol-Explorer. Over 35,000 compounds of low molecular weight were found in the gold standard database for natural compounds DNP. This number includes polyphenols present in non-food natural sources (non-crop plants, microbes, and other natural sources).

Most bioinformatics and data mining tools are not equipped *a priori* with chemical structure query functionalities which is normally a requirement and task of computational chemistry. The polyphenol list generated in the data mining step had to be matched to a database using identifiers which could be used to query protein interaction databases. CHEBI uses this feature but only about 1,300 compounds were found in this database. The number of polyphenols interacting with proteins further reduced the list to 1% of the initial catalogue. This low number reflects the lack of molecular studies on polyphenols. The PDB, a database of macromolecules with over 130 k entries (protein and nucleic acids, including small molecule complexes) was searched for crystal structures of polyphenol-protein complexes but only a small number was found. For example, quercetin was predicted to interact with 2,500 proteins based on the interaction databases but only 25 quercetin-protein complexes were found in the PDB. That is, the polyphenol literature describes levels of polyphenols in foods and biofluids and their biological effects in *in vitro* and *in vivo* studies, while very few studies analyse polyphenol-protein interactions. The lack of biochemical and structural characterization limit mechanistic understandings of their role in health and disease.

Data mining of existing public data demonstrated that most research was conducted on a few polyphenol ‘prima donnas’ and for which many benefits have been proposed^14^. These include (i) quercetin, the flavonoid present in apple and other fruits and vegetables, (ii) resveratrol, the stilbene produced in grapes after fungal or bacterial attack, (iii) genistein, the isoflavone present in beans and pulses such as soy, and (iv) coumestrol present in soy and legumes. Although searches yielded thousands of polyphenolic compounds, a larger number may be present because of reduction, oxidation, hydrolysis, phase I and II metabolic reactions in different organs and by gut microbiota. As polyphenol bioavailability is often poor, it is probable that biological activity may also be due to unknown and rapidly metabolized polyphenol intermediates or end-products. Characterizing the human polyphenol metabolome^5,6^ is of high relevance for understanding how inter-individual variability results from polyphenol-rich foods consumption and their effects on metabolism and consequent benefits for health^36^.

The polyphenol-protein interactome provides a systems-view of a wide variety of biochemical processes affected by these compounds, from central metabolism to signalling events. A number of proteins enriched in polyphenol interactions were cytochrome P450s (CYPs), carbonic anhydrases (CAs), aldehyde dehydrogenases (ALDHs), UDP-glucuronyl transferases (UGTs) and aldo-keto reductases (AKRs)^29^ which are responsible for detoxification. CYPs scored the highest in enrichment from all polyphenol-interacting proteins. CYPs account for ~75% of drug metabolism enzymes^37^, and of the 57 known human CYPs^38^, 5 are involved in ~95% of the drug metabolism reactions. By oxidizing substrates, CYPs help in the deactivation of many drugs and xenobiotics. Polyphenols have been described to interact and inhibit CYPs with mechanisms similar to that of single-target drugs^39^. Flavonoids induce drug metabolism enzymes such as NAD(P)H:quinone oxidoreductase-1 (NQO1), glutathione S-transferases (GSTs), aldo-ketoreductases (AKRs), and glutathione (GSH) biosynthetic enzymes (such as glutamate-cysteine ligase) resulting in scavenging of reactive oxygen species (ROS). The induction of these pathways is used to label polyphenols as indirect antioxidants. Some polyphenols are able to increase nuclear factor (erythroid-derived 2)-like 2 (Nrf2) activity, leading to the induction of its target genes which are involved in preventing oxidative damage^40,41^.

Membrane transport proteins such as ATP-binding cassette transporters (ABCs) and solute carrier family (SLCs) were found to interact with many polyphenols. The gut absorbs polyphenols (phase 0) by means of transporters, which modulate bioavailability of bioactive food ingredients and drugs. The same transporters are also responsible for eliminating potential detoxification products (phase III metabolism)^42^.

Nuclear receptor agonists and antagonists are commonly used as drugs for diabetes, and cancer^31^. Many polyphenols were found to interact with nuclear receptors such as peroxisome proliferator activator receptors (PPARs) and ERs. Certain nuclear receptors act as sensors of endobiotics and xenobiotics often through transporters^42^ and biotransformation reactions (phases I and II) mediated by CYPs. G-protein coupled receptors (GPCRs) are also therapeutic targets that interact with polyphenols. These transmembrane receptors comprise a large family of proteins involved in a variety of physiological roles. In particular, the metabolite-sensing GPCRs (including GPR43, GPR41, GPR109A, GPR120, GPR40) bind to various dietary metabolites produced in the gut and transmit signals of immune and metabolic relevance^43^.

Kinases are also major targets of polyphenols because they have the potential to bind to ATP-binding sites thereby modulating action of MAP kinase, phosphoinositide 3-kinase (PI 3-kinase), Akt/protein kinase B (Akt/PKB), tyrosine kinases, and protein kinase C (PKC) pathways. Inhibiting or stimulating these pathways influence phosphorylation events and modulation of gene expression^8^. The fact that various polyphenols interact with cyclin-dependent kinases (CDKs) highlights their potential control of cell cycle events, including cell proliferation and cancer development^44^. Major proteins involved in diabetes, such as AMPK, PPARs, dipeptidyl peptidase 4 (DDP-4), and others^30,34,45^ have been reported to interact with polyphenols, suggesting a potential therapeutic application for natural bioactives in the modulation of metabolic diseases.

In addition to the analysis of protein classes, pathway enrichment is a common strategy to interpret gene lists from large-scale analyses by mapping them to pathways^46^. The polyphenol-interacting proteins identified in this study were used to perform pathway enrichment using KEGG’s classification of pathways. The enrichment covered multiple processes for most polyphenol classes and pathway categories, indicating pleotropic metabolic activities and functions, signalling, and human diseases irrespective of polyphenol class. The relatively high coverage of pathways by flavonoids, phenolic acids, and other polyphenols was unexpected. Xenobiotic degradation and metabolism as well as oxidative pathways were enriched across all polyphenols classes. For example, flavonoids have a high coverage for nitrogen and ascorbate metabolism, which may be related to their antioxidant properties (reactive oxygen scavenging and nitrogen radical scavenging), in which quercetin is particularly efficient^47^. Flavonoids, and also other polyphenols, were highly represented in type 2 diabetes pathways and specifically for resveratrol, quercetin, epigallocatechin gallate^48^. Citrus fruit was used as a model to assess potential overlapping protein interactions that may occur when consuming a complex polyphenol-rich food where different components alter expression of genetic information and are also transformed through metabolic machinery. The polyphenols in grapefruit typified the highly overlapping effects on a wide variety of pathways.

The protein interactome of other bioactives (e.g., micronutrients) and an example drug (metformin) were used for comparison of the coverage found in analysis of the polyphenol interactome. These two types of bioactives yielded more restricted interactomes with more specific pathway functionalities. Pathways related to the known anti-diabetic drug metformin^49^ were particularly scarce compared to polyphenols, a result consistent with synthetic molecules designed to specifically interact with one protein target. Systems-based approaches for identifying multiple targets (e.g., polypharmacology) across interrelated networks may be an alternative strategy for disease management or health promotion, in particular in multifactorial diseases, as CMDs^50,51^. Low-effect promiscuous bioactives that interact with multiple proteins across many pathways in a concerted way may be more effective^52^, assuming that optimal doses can be found for different individuals. Based on the reported associations with proteins, polyphenols are multi-factorial activators modulating different pathways, some of which modulating CMDs and other diseases.

### Limitations

This study relies on publically available databases for (i) polyphenols, (ii) polyphenol content in foods, (iii) polyphenol-protein interactions, and (iv) polyphenol-induced pathways. The polyphenol functionalities are thus limited to published studies. Our analysis demonstrated that the current literature is highly skewed by a limited number of polyphenols, which have been analysed primarily for their effect on gene expression and signalling, regulators, catalytic enzymes, and pharmacological targets. Other potential interactions, such as direct high- or low-affinity binding to DNA or RNA have not been analysed. The polyphenol-protein interactome described here was based on metabolic and signalling proteins. Polyphenol concentration-driven effects and bioavailability effects were not taken into account in pathway enrichment.

Nevertheless, this study showed the status of polyphenol research with all its complexities and limitations. The lack of information on human metabolism of many polyphenols, in particular gut microbial biotransformations, as well as the lack of knowledge on molecular interactions (i.e., 3D-protein structure with individual polyphenols) suggests that substantial research needs to be done to understand the mechanistic intricacies with potential health benefits of polyphenols.

### Strengths

This study (i) combined data integrated from over 25 different databases on polyphenol structures and interactions, (ii) took into account polyphenols and their known human metabolites, and (iii) provided a global systems-overview of current polyphenol research based on protein interactions and pathway mapping incorporating knowledge of polyphenol biochemistry and metabolism, pharmacology, and potential health benefits with a particular focus on CMDs. The overlap of functionalities between polyphenol (sub)classes, as well as the wide range of pathways involved suggest that polyphenols may have mild but widespread effects on metabolism, a concept distinct from known effects of “single” target drugs. The polyphenol-protein interactome can be explored for other diseases and/or biological phenomena and serve as starting point to design new studies.

### Summary

Polyphenols may be used in combination with drugs to modulate drug oral bioavailability and/or prevent multidrug resistance, at least in certain instances. However, metabolism and bioactivity of polyphenols is highly complex and involves participation of multiple organs. While many *in vitro* studies have been conducted with polyphenols, reproducibility of *in vivo* studies has proven challenging and often contradictory. Conflicting results occur because of (i) variability of doses including use of non-physiological and very high doses, (ii) testing of pure polyphenols compared to polyphenol-rich foods which ignores the presence of other potential bioactives, (iii) variation in polyphenol contents in foods, (iv) the use of *in vitro* models that lack inter-cell and organ interactions, and (v) poor bioavailability. Human studies are also affected by inter-individual variability^36^ in environment (unmeasured confounders in diet and environment) and host and microbial genetic differences that are involved in the biotransformation and absorption of polyphenols and metabolites.

## Methods

### Polyphenols list

Over 250,000 entries with fully defined chemical structures from DNP^2^ 25.1 (CRC) were imported into a local JChem (ChemAxon) chemical structure database. The database was then queried for the presence of any of the 43 defined polyphenol substructures, obtained from 31 defined sub-classes of polyphenols according to Phenol-Explorer (version 3.6 April 2015)^21^, Fig. 1. Default settings for stereochemistry and double bond isomery were applied. Molecules with molecular mass >1200 (ca. 900), contained nitrogen (ca. 6100, mainly alkaloids), and steroids (80) were removed to yield 36,064 unique polyphenols present in DNP. The process was designed as a KNIME workflow^53^. Chemical Entities of Biological Interest (CHEBI) structural entries (90,129) were identified in the DNP but 6,827 did not have chemical structures and were thus removed leaving 83,302 compounds. Chemical structures from both CHEBI and DNP polyphenol lists were standardized based on the following scheme: (i) remove fragments, (ii) neutralize, (iii) remove explicit hydrogens, (iv) tautomerize, and (v) aromatize. Finally, unique SMILES strings were generated. The list comparison was done by matching SMILES (Simplified molecular-input line-entry system) strings using strict stereochemistry criteria. Since many public chemical databases do not describe stereochemistry of compounds, some matches were likely not found. However, erroneous assignment of CHEBI identifiers to polyphenols was avoided. CHEBI identifiers were assigned to 1179 DNP polyphenols, which was 3.2% of the total list. All chemical structure manipulations and data analysis were performed with JChem nodes in KNIME.

The list of CHEBI identified DNP polyphenols was merged with ‘polyphenol metabolites’ and ‘polyphenol compounds’ available from Polyphenol-Explorer^21^, leading to the final list.

### Polyphenol-protein interactions list

To query small molecule-protein interaction databases, the polyphenol list was searched in CHEBI database^54^, containing ~100,000 entries, for corresponding CHEBI ID’s based on standardised SMILES. Polyphenol-protein interactions were mined from the STITCH^22^, Pathway Commons^23^ and BindingDB^24^ databases, which collect information from multiple and often redundant databases, using name and CHEBI ID. Depending on the database, the term ‘interaction’ can either refer to direct binding (most interactions in STITCH and Pathway Commons and all interactions in BindingDB) or indirect interactions. STITCH also details the type of evidence (i.e. experimental, database, prediction, text mining) and the confidence score associated to each interaction listed. The confidence score ranged from 0.15 (low evidence) to 0.9 (highest evidence). Interactions based solely on text mining evidence or with confidence score <0.9 were excluded.

The proteins obtained from these searches were analysed with DAVID^55^ tools for Functional Annotation Clustering using InterPro (Protein sequence analysis & classification)^26^ for protein clustering and classification. Enrichment scores >3 were considered.

The quercetin and metabolites – protein interactomes were constructed as a network using Cytoscape v.4.3.0^56^. The organic micronutrient-protein interactome was mined from STITCH, excluding interactions based solely on text mining and those with evidence scores <0.7. The metformin-protein interactome was obtained using the same methodology but excluding interactions with evidence score <0.9.

### Functional enrichment analysis

Pathway enrichment analysis was performed with R packages HTSAnalyzeR for KEGG^57^ to evaluate polyphenol involvement in biological pathways through their interactome. Benjamini-Hochberg (BH) adjusted *p*-values below 0.1 and pathway coverage above 10% were considered significant. Significant KEGG pathways were mapped to their categories (e.g. metabolism, genetic information processing) and subcategories (e.g. carbohydrate, energy, lipid metabolism) as detailed by the pathway ontology from KEGG. Bar charts were used to represent significant pathway enrichments in which distance between pathways is related to pathway gene set similarity.

A food-based analysis was also conducted comparing the polyphenol composition and interactome of grapefruit (*Citrus* × *paradisi*), lemon (*Citrus limon*), lime (*Citrus* × *aurantifolia*), orange (*Citrus* × *sinensis*), tangerine (*Citrus* × *tangerina*) and pomelo (*Citrus maxima*) fruits and pure citrus fruit juices. The polyphenol composition of these citrus was extracted from Phenol-Explorer and all polyphenols were considered irrespective of their concentration in the fruit. Using the polyphenol-protein interactome, interacting proteins were identified and KEGG Metabolism pathways were attributed.

## Electronic supplementary material

Supplementary Materials

Table 1S
