# Supplementary material for: A computationally driven analysis of the polyphenol-protein interactome
Source: Sci Rep. 2018 Feb 2;8:2232. doi: 10.1038/s41598-018-20625-5 (PMC5797150; doi:10.1038/s41598-018-20625-5)
Supplement: Supplementary file 1 — Supplementary Materials [file 41598_2018_20625_MOESM1_ESM.pdf]

A computationally driven analysis of the polyphenol-protein interactome

Sébastien Lacroix [1], Jasna Klicic Badoux [2], Marie-Pier Scott-Boyer [1], Silvia Parolo [1], Alice Matone [1], Corrado Priami [1,3], Melissa J. Morine [1], Jim Kaput [2], Sofia Moco [2]*

[1] The Microsoft Research – University of Trento Centre for Computational and Systems Biology (COSBI), Rovereto (TN), Italy.

[2] Nestle Institute of Health Sciences, Lausanne, Switzerland.

[3] Department of Computer Science, University of Pisa, Pisa (PI), Italy.

* Correspondence and requests for materials should be addressed to S.M. (sofia.moco@rd.nestle.com)

Supplementary Figures

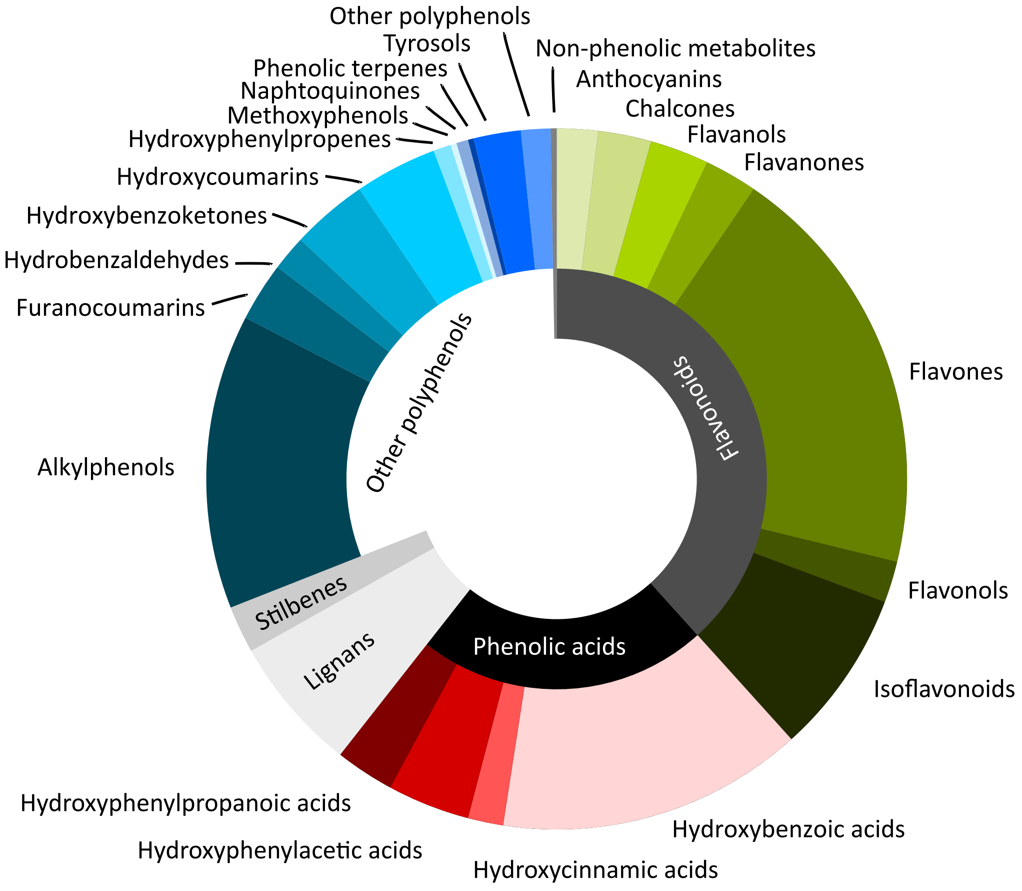

Figure 1S. Classification of protein interacting polyphenols according to classes and sub-classes.

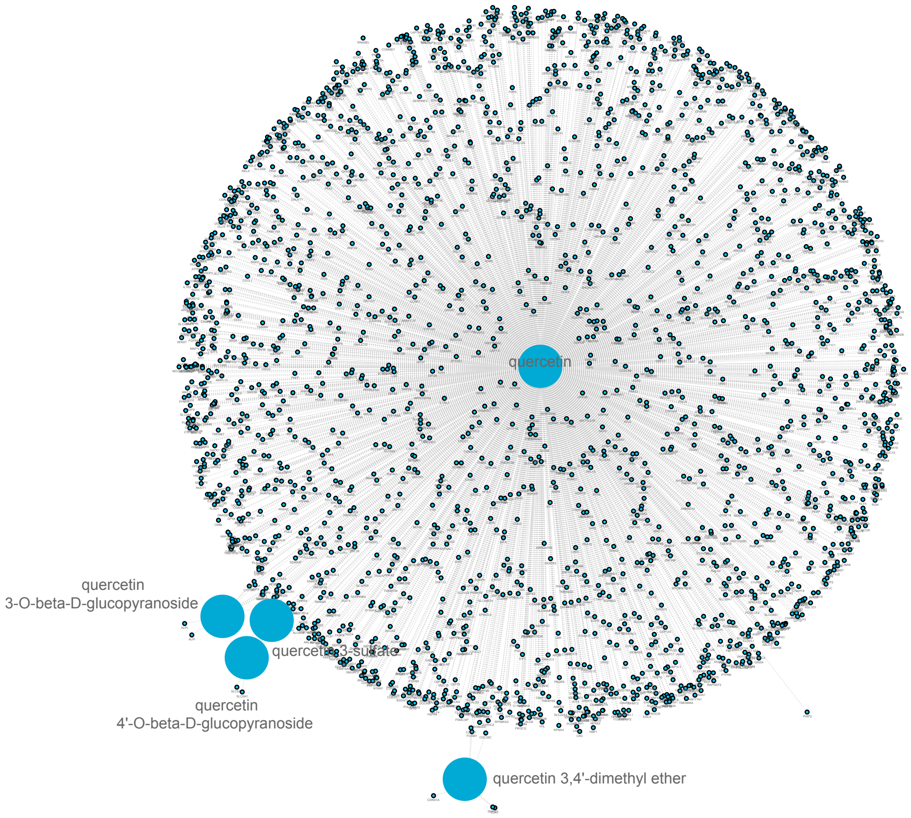

Figure 2S. Polyphenol-protein interactions network for quercetin (2500 interactions) and derivatives: quercetin 3-O-beta-D-glucopyranoside, quercetin 4’-O-beta-D-glucopyranoside, qurecetin 3-sulfate and quercetin- 3,4’-dimethyl ether (46 interactions).

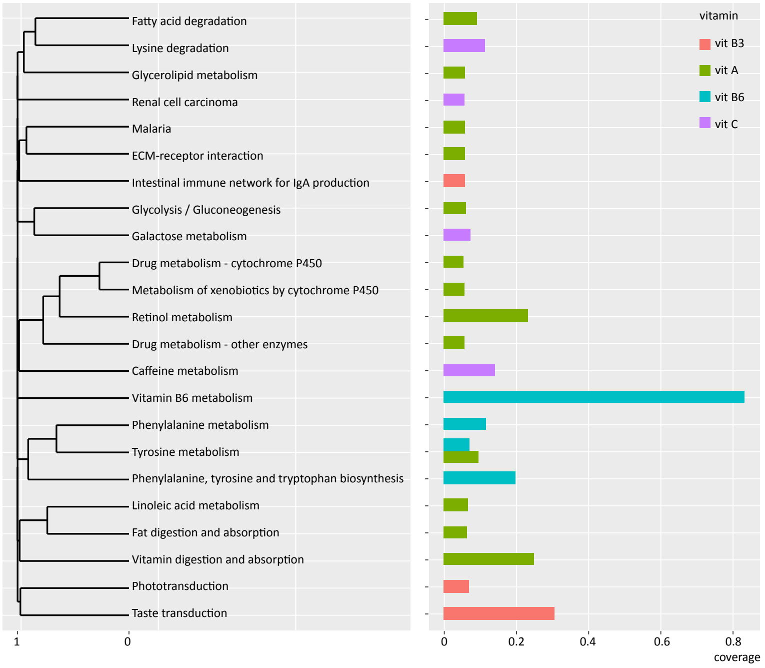

Figure 3S. KEGG pathway enrichment coverage from four organic micronutrients: vitamin B3 (vit B3), vitamin A (vit A), vitamin B6 (vit B6), and vitamin C (vit C) obtained from their protein interactome according to STITCH, and clustered according to p-value.

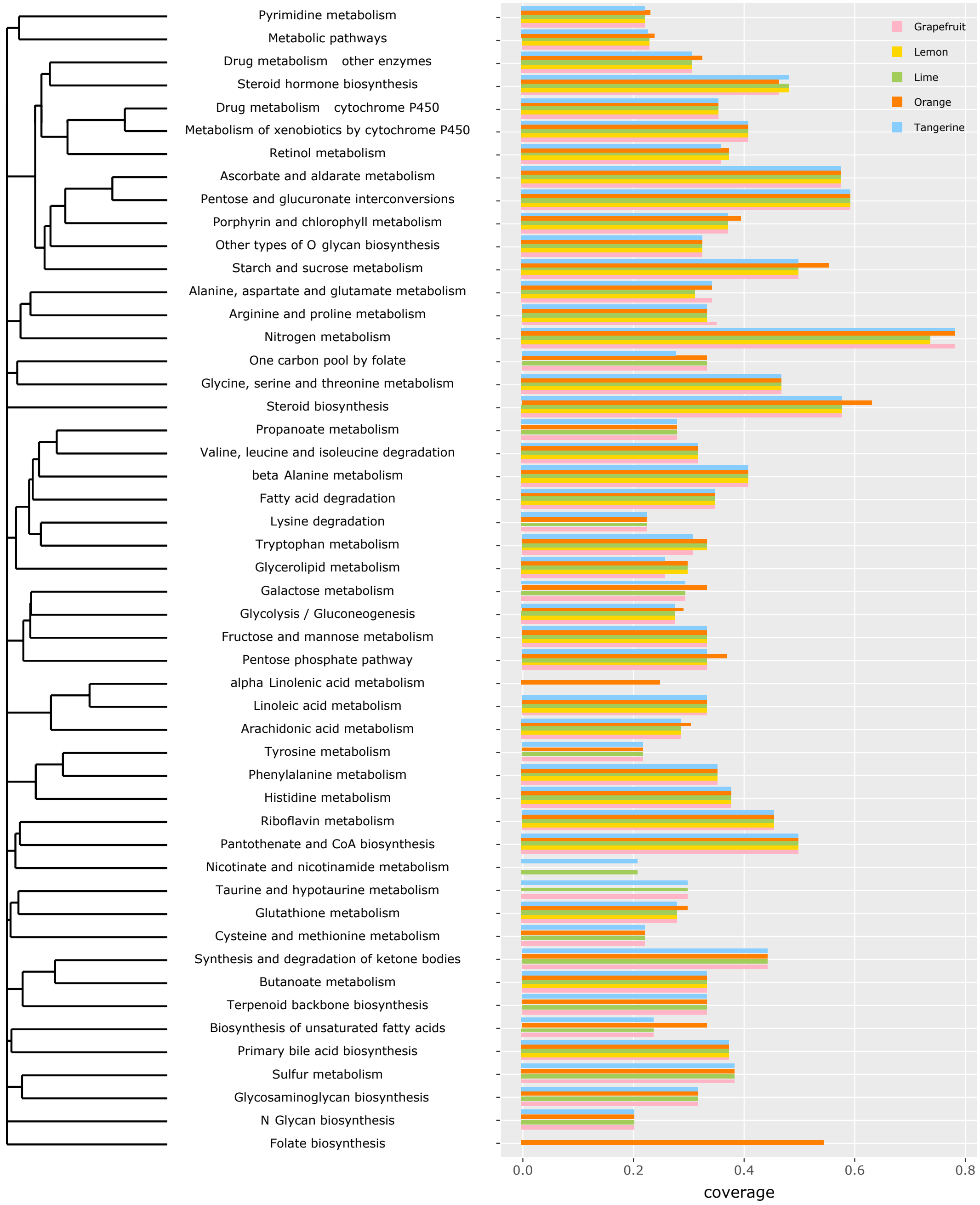

Figure 4S. Dendogram of KEGG's metabolism pathways enriched for grapefruit, lemon, lime, orange, and tangerine (with coverage > 0.2, clustered according to p-value).
